# Agglomeration of Productive Services, Industrial Structure Upgrading and Green Total Factor Productivity: An Empirical Analysis Based on 68 Prefectural-Level-and-Above Cities in the Yellow River Basin of China

**DOI:** 10.3390/ijerph191811643

**Published:** 2022-09-15

**Authors:** Xu Dong, Yang Chen, Qinqin Zhuang, Yali Yang, Xiaomeng Zhao

**Affiliations:** 1 School of Economics, Zhengzhou University of Aeronautics, Zhengzhou 450046, China; 2 Institute of Quantitative and Technical Economics, Chinese Academy of Social Sciences, Beijing 100732, China; 3 School of Information Management, Zhengzhou University of Aeronautics, Zhengzhou 450046, China; 4 School of Economics and Business Administration, Central China Normal University, Wuhan 430079, China

**Keywords:** green total factor productivity, agglomeration of productive services, industrial-structure upgrading, the Yellow River Basin, lean green, sustainability

## Abstract

Improving green total factor productivity (GTFP) is the inherent requirement for practicing the philosophy of green development and achieving regional high-quality development. Based on panel data for 68 prefectural-level-and-above cities in the Yellow River Basin of China from 2006 to 2019, we measured their GTFPs and degrees of productive-services agglomeration using the non-radial directional distance function and industrial agglomeration index formulas, respectively. Furthermore, we empirically investigated the interactive relationship between agglomeration of productive services, industrial-structure upgrading, and GTFP using the dual fixed-effects model, the mediating-effect model, and the moderating-effect model. The findings were as follows. (1) Both specialized and diversified agglomeration of productive services significantly improved the GTFPs of cities in the Yellow River Basin, and the promoting effect of specialized agglomeration was stronger than that of diversified agglomeration. (2) The diversified agglomeration of productive services (hereinafter referred to as diversified agglomeration) made a significant contribution to GTFP in all sample cities of the Yellow River Basin, while the specialized agglomeration of productive services (hereinafter referred to as specialized agglomeration) only significantly improved GTFP in the upstream cities and had no significant effect on the midstream and downstream cities. (3) When examined according to city size, specialized agglomeration was found to have a positive impact on the GTFPs of small and medium-sized cities in the Yellow River Basin but a non-significant negative impact on large cities, while the effect of diversified agglomeration on GTFP was found not to be significant. (4) Industrial-structure upgrading played partially mediating and negative moderating roles in the process of specialized agglomeration affecting the GTFPs of cities in the Yellow River Basin, but it did not become a mediating channel and moderating factor that influenced diversified agglomeration in relation to GTFP.

## 1. Introduction

Since the reform and opening up, China’s rapid economic development has created a world-renowned “China miracle”, but the extensive development mode has also brought about serious energy consumption and pollution emissions, such that China’s sustainable economic development faces serious challenges [1,2]. Total factor productivity (TFP) is an important indicator used to measure the quality of economic development. Standing in the new historical position of China’s development, the 19th National Congress of the Communist Party of China emphasized that “we should promote changes in the quality, efficiency and driving force of economic development, and improve total factor productivity”. Furthermore, the National 14th Five-Year Plan pointed out that “we should promote the comprehensive transformation of economic and social development, and build a beautiful China”. Accordingly, against the background of increasingly tightening resource and environmental constraints, improving economic efficiency, reducing pollution emissions, and promoting intensive growth have become inevitable courses of action for China in the current and even in the future period. The aim, essentially, is to improve green total factor productivity. As the basic spatial vector leading national competition, cities, under the conditions of resource and environment constraints and the shock of COVID-19 pandemic, how to promote GTFP improvement is crucial for a country to cultivate competitive advantages and new growth points [3,4].

Different from traditional TFP, when measuring GTFP, resource and environmental factors are taken into account, so GTFP can reflect the quality of economic development more scientifically and reasonably [5]. In this context, many scholars have measured China’s GTFP at different spatial scales, such as enterprise, industry, and regional scales, and have systematically explored the paths of its enhancement [6,7]. It has been found that environmental regulations, levels of financial development, public expenditure policies, industrial agglomeration, technological innovation, and foreign direct investment are likely to affect GTFP [8,9,10,11,12,13,14]. However, there is no unanimous academic conclusion as to whether these factors can effectively contribute to GTFP.

With the increasing prominence of ecological and environmental problems, the question of how to achieve sustainable economic development has received widespread attention in academia. Most Chinese scholars have conducted research from the perspective of green finance and have found that green finance can provide financial support for the development of environmental protection and other related industries, promote industrial-structure upgrading, and thus contribute to sustainable economic development [15]. A study by Han et al. (2019) argued that with the support of green finance, Chinese industrial are shifting towards greening, rationalization, and high-end development, thus ultimately contributing to sustainable economic development [16]. The relevant Western literature has focused on the impact of technological progress on sustainable development and has achieved fruitful outcomes. Studies have shown that technological progress is a decisive factor in achieving sustainable development [17] and that factors such as green innovation and external-knowledge spillover make significant contributions to enhancing the sustainable business capacities of enterprises and achieving sustainable economic development [18]. Fan et al. (2015) and Davou et al. (2022) empirically tested mechanisms of green technological progress in sustainable development using sample data from China and Malaysia and found that they can effectively mitigate the conflict between industrial growth and environmental protection by reducing carbon emissions, thus contributing to regional sustainable economic development [19,20]. Additionally, other scholars have focused on the impact of lean processes on sustainable performance, arguing that the relationship between the two is dynamic and changing. It is only when the relationship between the two is in a harmonious stage that they will have a positive impact on sustainable business performance [21]. Hartini et al. (2020) improved the established sustainability-performance-measurement method based on the concept of lean and sustainable development and comprehensively measured the sustainability performance of the furniture industry in Indonesia [22]. Since this method has a more scientific basis, it will be applied to more sectors in the future to measure sustainable performance in different industries. Teixeira et al. (2022) assessed the impact of lean and green (LG) practices on the competitive advantages (CAs) of organizations, and the results demonstrated a positive impact of LG practices on sustainable development [23]. The popularity and depth of the philosophy of sustainable development requires human beings to always pay attention to environmental protection in the process of economic development, which also highlights the practical necessity of enhancing green total factor productivity.

As areas of modern service industry that are detached from the manufacturing industry and which have developed independently, productive services are characterized by significant economies of scale, high technology utilization and knowledge intensity, low pollution, and energy consumption. The spatial agglomeration of productive services is gradually becoming an important force in promoting China’s economic growth. Some scholars have studied the growth effect of TFP caused by the externalities of productive-services agglomeration, and the results show that the agglomeration of productive services can promote manufacturing-technology innovation through the effects of knowledge spillover, technology diffusion, economies of scale, and competition, thus contributing to TFP [24]. With the philosophy of green development gaining in popularity, the green development effect of productive-services agglomeration has gradually become a new research hotspot, and the impact of productive-services agglomeration on GTFP has received wide attention. Some studies have found that moderate agglomeration of productive services can significantly reduce urban-haze pollution and improve GTFP [25,26]. In addition, the GTFP growth effect of productive-services agglomeration varies according to the size, industrial structure, and administrative level of cities [27]. In terms of the transmission mechanism, many scholars believe that the agglomeration of productive services has a “structural-dividend” effect of promoting industrial-structure upgrading, which can effectively reduce energy consumption and pollution emissions per unit of output, alleviate regional resource and environmental constraints, and thus enhance GTFP [28,29,30]. Therefore, theoretically speaking, industrial-structure upgrading plays a non-negligible role in the process of GTFP enhancement by the agglomeration of productive services.

The Yellow River Basin is an important ecological barrier and economic region in China and plays a very important role in China’s economic and social development and eco-safety [31,32]. For a long time, due to a large number of resource-based cities, the problem of energy dependence for industries in the Yellow River Basin has been prominent. The economic-development model of over-reliance on resource development has caused most cities in the basin to face problems, such as high resource loads, ecological damage, and serious environmental pollution, posing a great challenge to sustainable economic development [33]. From the perspective of industrial structure, the Yellow River Basin is dominated by low-end industries characterized by rough and predatory development. Compared with the national average, the proportion of services is low. The insufficient supply of high-end factors of production and the weak capacity for scientific and technological innovation result in the low efficiency of green economic growth and inadequate endogenous power for industrial green transformation and upgrading [34]. In order to alleviate the conflict between economic development and ecological protection, in September 2019, China proposed to implement a major national strategy for ecological conservation and high-quality development in the Yellow River Basin. Subsequently, how to coordinate the relationship between economic development and environmental protection in the Yellow River Basin in order to achieve its high-quality economic growth has rapidly become a hot issue in academia [35,36]. As mentioned above, the agglomeration of productive services can reduce pollution emissions and improve green economic efficiency by promoting technological innovation and diffusion, optimizing industrial structure, and promoting the recycling of resource factors. Therefore, exploring the green economic growth effect of productive-services agglomeration probably provides a new reference for optimizing the industrial layout of the Yellow River Basin and promoting its ecological protection and high-quality development. In this context, we took 68 prefectural-level-and-above cities in the Yellow River Basin as samples to explore the relationship between the agglomeration of productive services and GTFP and to analyze the effect of the former on the latter from the perspective of industrial-structure upgrading.

The main possible contributions of this study are two-fold. The first is an academic contribution. By analyzing the theoretical mechanism of productive-services agglomeration affecting GTFP from the perspective of industrial-structure upgrading and provide empirical evidence with a sample of cities in the Yellow River Basin, we enrich the existing theoretical and empirical research results. The second contribution is a practical one, the study having implications for the industrial transformation and upgrading of cities in the Yellow River Basin, which play important roles in supporting the whole Yellow River Basin, enabling a resolution of the conflict between economic development and resource and environmental constraints and the achievement of high-quality development.

The rest of this paper is organized as follows. In Section 2, we conduct a theoretical analysis of the interaction between productive-services agglomeration, industrial-structure upgrading, and GTFP by combing the relevant literature and proposing research hypotheses. Section 3 presents the empirical research design for describing the model setting, variable selection, and data sources. Section 4 provides the empirical results and discusses them, including benchmark regression results, robustness tests, heterogeneity analysis, and impact mechanism tests. Finally, we summarize the study conclusions and present corresponding policy implications and future prospects in Section 5.

## 2. Theoretical Analysis and Research Hypotheses

### 2.1. Agglomeration of Productive Services and GTFP

Firstly, specialized agglomeration promotes enterprise innovation through economies of scale and knowledge spillovers, which, in turn, affect GTFP. Marshall (1890) argued that specialized agglomeration could promote the sharing of labor and intermediate input markets among enterprises in the same industry [37]. The economies-of-scale effect caused by sharing mechanisms reduces the costs of enterprises in talent searching, operations and management, and production transactions, allowing more funds for technological research, and promotes the transformation of enterprises into innovation-driven green and efficient development modes, which, in turn, improves GTFP in the agglomeration area. The economies of scale formed by the specialized intermediate input market can also promote the effective integration of knowledge and technology and other advanced factors in the production process by strengthening the input–output linkage between upstream and downstream enterprises and refine the division of labor among enterprises, which will improve the allocation efficiency of factors of production among enterprises and ultimately promote the production efficiency of enterprises in the agglomeration area [38]. In addition to economies of scale, specialized agglomeration can also lead to intra-industrial technological innovation and knowledge-spillover effects [37]. In general, specialized agglomeration may exacerbate market competition among geographically adjacent enterprises, and enterprises of productive services are bound to provide higher-quality products through innovation in order to occupy a market share in a limited market space [39]. The differentiated competition among enterprises drives the level of technological innovation in industry. Formal and informal learning exchange organizations within industry facilitate the generation and dissemination of new knowledge and help promote green technology R&D, thereby increasing GTFP [40,41].

Secondly, corresponding to the externality of specialized agglomeration, Jacobs’ externality theory emphasizes that diversified agglomeration of enterprises in different industries is conducive to inter-firm technological exchange and cooperation [42], and the resulting inter-industry technological innovation and diffusion effects can improve GTFP. On the one hand, diversified agglomeration is helpful to the cross-industry flow of highly skilled talent, driving the cross-collision of knowledge, technology, and skills among complementary industries and triggering the diffusion and spillover of knowledge and technology among related industries. On the other hand, diversified agglomeration can also enable enterprises to obtain diversified intermediate service products through socialized networks and increase the selectivity of pollution emissions and environmental-management-outsourcing services, thus enabling them to purchase specialized and scaled pollution-management services [43], which is conducive to enhancing GTFP in the agglomeration area. Based on the above analysis, we propose the following research hypothesis:

**Hypothesis** **1** **(H1).** *Both specialized and diversified agglomeration of productive services can significantly contribute to GTFP in the Yellow River Basin.*

### 2.2. Industrial-Structure Upgrading and GTFP

As an important basis of economic growth, the economic effect of industrial-structure upgrading has been widely attended to by academics. Most scholars’ studies have been conducted from two perspectives of “increasing quantity” and “improving quality”. From the perspective of “increasing quantity”, enterprises can improve production tools through technological innovation in the process of industrial-structure upgrading, enhance the productivity of input factors and promote sustained economic growth [44]. Industrial-structure upgrading can also improve TFP through specialization in the division of labor and technology spillover [45]. Duarte and Restuccia (2020) found that the development of high-end productive services had a positive effect on TFP in a study of different industrial sectors [46]. From the perspective of “improving quality”, industrial-structure upgrading also plays a positive role in promoting GTFP. Gu et al. (2022) argued that industrial-structure upgrading has the effect of curbing pollutant emissions and significantly improves environmental quality [47]. In addition, the “structural dividend” released by industrial-structure upgrading can promote the emergence of clean industries and provide effective support to improve GTFP. Wang et al. (2018), Zhu et al. (2019), and Sun et al. (2022) have further maintained that industrial-structure upgrading can significantly improve urban GTFP in the context of environmental regulation, energy control, and low-carbon city construction [48,49,50]. Accordingly, we propose the following research hypothesis:

**Hypothesis** **2** **(H2).** *Industrial-structure upgrading has a promoting effect on GTFP in the Yellow River Basin.*

### 2.3. Agglomeration of Productive Services and Industrial-Structure Upgrading

From the perspective of specialized agglomeration, the influence of Marshall externalities on industrial-structure upgrading is mainly reflected in three aspects. First, productive services constitute an industry that requires a large amount of human capital investment. Its specialized agglomeration will strengthen human-capital investment and enhance the level of human-capital accumulation in the agglomeration area, facilitate the formation of learning and communication networks, contribute to the generation and diffusion of technical knowledge, improve the quality and added value of enterprise products, and promote the high-end transformation of productive services [51,52]. Second, specialized agglomeration leads to increase in the number and scale of homogeneous enterprises and to competition among enterprises becoming increasingly fierce. In order to gain advantages in homogeneous competition, enterprises will enlarge efforts of technological innovation and equipment replacement to reduce production costs and achieve differentiated operations, thus leading to the upgrading of industrial structure [53]. Third, specialized agglomeration can alleviate the asymmetry of market information, facilitate enterprises to provide timely, fast, and accurate production services required by other market players, and improve the efficiency of resource allocation. Under the influence of the market mechanism, the continuous refinement of the specialized division of labor and collaboration means that enterprises can focus more on process improvement and speed up product renewal, thus promoting the transformation and upgrading of industrial structure [54].

Similarly, the impact mechanism of diversified agglomeration on industrial-structure upgrading can be summarized in terms of the following three factors. First, diversified agglomeration strengthens the input–output linkage between upstream and downstream industries, promotes vertical and horizontal cooperative division of labor among enterprises in the industrial chain, reduces production costs, and improves the efficiency of collaboration between different sectors, thus promoting industrial-structure upgrading [55]. Second, diversified agglomeration expands market demand and scale, and the market puts forward higher requirements for industrial division of labor, pushing the specialized division of labor in productive services to further refine and deepen and forcing manufacturers to carry out technological innovation [56,57]. Third, diversified agglomeration is conducive to the deep integration of talent, capital, technology, and management experience among different industries through social networks, alleviating factor-market segmentation, improving factor-allocation efficiency, and realizing industrial transformation and upgrading [58].

In addition, some scholars are concerned that the agglomeration of productive services may be constrained by some factors in the promotion of industrial-structure upgrading [59]. For example, Wan and Li (2020) found that the cost of promoting industrial-structure upgrading is higher in areas with a level of low economic development and poor public infrastructure and that the agglomeration of producer services cannot promote or even inhibit the upgrading of local industrial structure [60]. According to Yu (2019), specialized and diversified agglomeration can only enhance enterprise productivity if a certain threshold is reached, and market demand for productive services in regions with large industry sizes and few types of industries is often characterized as “large but single”, which is more conducive to specialized agglomeration [61]. At present, the Yellow River Basin is dominated by resource-based industries and traditional manufacturing industries, with a relatively uniform industry type and the transformation and upgrading of industrial structure still in the primary stage. Therefore, specialized agglomeration is more in line with the practical needs of economic development and may play a leading role in the upgrading of industrial structure in the Yellow River Basin. Given the above analysis, the following research hypothesis is put forward:

**Hypothesis** **3** **(H3).** *Specialized agglomeration can significantly promote industrial-structure upgrading in the Yellow River Basin cities, while the effect of diversified agglomeration on industrial-structure upgrading may not be obvious.*

### 2.4. Agglomeration of Productive Services, Industrial-Structure Upgrading, and GTFP

According to the above analysis, it is clear that both specialized and diversified agglomeration of productive services can directly promote GTFP through agglomeration externalities. Meanwhile, some theoretical and empirical studies have shown that industrial-structure upgrading is an important inducement to promote regional GTFP [46,48,50]. Considering the influence of economic-development level and industrial type, specialized agglomeration can improve GTFP through industrial-structure upgrading, while this transmission path has not been confirmed in diversified agglomeration. In recent years, although the Yellow River Basin has also been actively pushing the transformation and upgrading of industrial structure, it has not yet jumped out of the traditional development pattern and lacks the impetus for industrial ecological transformation. Due to the lack of policy guidance and scientific planning, many industrial-agglomeration areas in the Yellow River Basin suffer from unreasonable industrial structures and low development levels [62]. Therefore, industrial-structure upgrading may have adverse intervention effects in the process of productive-services agglomeration, affecting GTFP in the Yellow River Basin. In accordance with this, the following two research hypotheses are presented:

**Hypothesis** **4** **(H4).** *Specialized agglomeration promotes the GTFPs of cities in the Yellow River Basin through industrial-structure upgrading, while the influence mechanism of diversified agglomeration is not apparent.*

**Hypothesis** **5** **(H5).** *Industrial-structure upgrading has a negative moderating effect on the process of productive-services agglomeration, affecting GTFP in the Yellow River Basin cities.*

## 3. Empirical Research Designs

### 3.1. Models

Firstly, we used a dual fixed-effects model to examine the direct impact of productive-services agglomeration on GTFP in cities in the Yellow River Basin. To mitigate possible heteroskedasticity, the variables were taken in logarithmic form and the model was set as follows:

(1) $$\ln gtfp_{it}=\alpha_{0}+\alpha_{1}\ln spe_{it}+\alpha_{2}\ln div_{it}+\sum \alpha_{j}\ln controls_{jt}+\mu_{i}+\lambda_{t}+\varepsilon_{it}$$

where i denotes city and t denotes year. spe and div denote the specialized agglomeration and diversified agglomeration of productive services, respectively. Vector controls are a series of control variables, including technology-input intensity, foreign direct investment, financial-development level, information infrastructure, and economic-development level. $\mu_{i}$, $\lambda_{t}$, and $\varepsilon_{it}$ denote the city fixed effect, the time fixed effect, and the random disturbance term, respectively.

Secondly, according to the theoretical analysis, the agglomeration of productive services may affect the GTFP of cities in the Yellow River Basin through industrial-structure upgrading. We used the mediating-effect model to test this transmission mechanism. Referring to the method proposed by Wen and Ye (2014) [63], Equations (2) and (3) were constructed by combining Equation (1) as follows:

(2) $$\ln ind_{it}=\beta_{0}+\beta_{1}\ln agg_{it}+\sum \beta_{j}\ln controls_{jt}+\mu_{i}+\lambda_{t}+\varepsilon_{it}$$

(3) $$\ln gtfp_{it}=\gamma_{0}+\gamma_{1}\ln agg_{it}+\gamma_{2}\ln ind_{it}+\sum \gamma_{j}\ln controls_{jt}+\mu_{i}+\lambda_{t}+\varepsilon_{it}$$

In Equations (2) and (3), ind is the mediating variable, namely, industrial-structure upgrading; agg is the agglomeration index of productive services, including the specialized agglomeration index and diversified agglomeration index; and the other variables have the same meanings as above.

Additionally, in order to test whether industrial-structure upgrading intervenes in the process of productive-services agglomeration affecting the GTFP of cities in the Yellow River Basin, the following moderating-effect model was set:

(4) $$\ln gtfp_{it}=\delta_{0}+\delta_{1}\ln spe_{it}+\delta_{2}\ln ind_{it}+\delta_{3}\ln spe_{it}\times \ln ind_{it}+\sum \delta_{j}\ln controls_{jt}+\mu_{i}+\lambda_{t}+\varepsilon_{it}$$

(5) $$\ln gtfp_{it}=\theta_{0}+\theta_{1}\ln div_{it}+\theta_{2}\ln ind_{it}+\theta_{3}\ln div_{it}\times \ln ind_{it}+\sum \theta_{j}\ln controls_{jt}+\mu_{i}+\lambda_{t}+\varepsilon_{it}$$

In Equations (4) and (5), lnspe×lnind and lndiv×lnind represent the interaction terms of specialized agglomeration, diversified agglomeration, and industrial-structure upgrading, respectively. Parameters $\delta_{3}$ and $\theta_{3}$ reflect the magnitude of the moderating effect of industrial-structure upgrading, and the meanings of the other variables are the same as above.

### 3.2. Variables and Data

#### 3.2.1. Dependent Variable

The dependent variable is the city green total factor productivity index (gtfp). Taking cities in the Yellow River Basin as decision-making units (DMUs) and referring to the approach of Lin and Tan (2019), the non-radial directional-distance function (NDDF) is used to measure their GTFPs [64]. The detailed steps are as follows. Firstly, the input factors for each DMU are identified as capital (K), labor (L), and energy (E), and the desired output is GDP, while industrial wastewater (W), sulfur dioxide (S), and industrial dust (D) are selected as undesired outputs. Secondly, the “multi-input and multi-output” production function for each DMU is set, and the NDDF function is defined. Then, the optimal relaxation variable $\beta^{*}$ is obtained by setting the weight vector $W^{T}$ and direction vector G to solve the NDDF function in linear programming. When determining the weight vector, a weight of 1/3 is usually assigned to each of the input factors and desired and undesired outputs, based on the principle that the variables are equally important [65], i.e., $W^{T}=\left(1/9,\;1/9,\;1/9,\;1/3,\;1/9,\;1/9,\;1/9\right)$. Since the substitution effect of labor and capital for energy affects the energy-environmental performance to some extent, this leads to an inability to obtain a true picture of the extent of energy wastage in the production process. Therefore, it is necessary to decompose labor and capital inefficiently and set their weights to 0. Then, the weight vector is $W^{T}=\left(0,\;0,\;1/3,\;1/3,\;1/9,\;1/9,\;1/9\right)$, and the corresponding direction vector is $G=\left(0,\;0,-E,-GDP,-W,-S,-D\right)$. On this basis, the optimal solution $\beta^{*}=\left(\beta_{E}^{*},\beta_{GDP}^{*},\beta_{W}^{*},\beta_{S}^{*},\beta_{D}^{*}\right)$ can be calculated. Finally, the GTFP of city *i* in period *t* is constructed according to the optimal solution as follows:

(6) $$gtfp_{it}=\frac{1}{2}\;\left[\frac{\left(E_{it}-\beta_{E}^{*}E_{it}\right)/\left(GDP_{it}+\beta_{GDP}^{*}GDP_{it}\right)}{E_{it}/GDP_{it}}\right]+\frac{1}{2}\;\left[\frac{1}{3}\underset{U=W,S,D}{\sum }\frac{\left(U_{it}-\beta_{U}^{*}U_{it}\right)/\left(GDP_{it}+\beta_{GDP}^{*}GDP_{it}\right)}{U_{it}/GDP_{it}}\right]$$

#### 3.2.2. Independent Variables

According to the industrial classification for national economic activities of the People’s Republic of China (GB/T 4754-2017), we identified the following five industries as productive services in this paper: (1) information transmission, software, and information technology; (2) financial intermediation; (3) leasing and business services; (4) transport, storage, and post; and (5) scientific research and technical services. Since most scholars have studied industrial agglomeration from the perspective of specialization and diversification, we further classified the agglomeration of productive services into specialized agglomeration (*spe*) and diversified agglomeration (*div*).

According to Ezcurra et al. (2006) [66], the specialized agglomeration index is calculated as:

(7) $$spe_{it}=\sum \left|\frac{E_{is}}{E_{i}}-\frac{E_{s\prime }}{E^{\prime }}\right|$$

where $E_{is}$ represents the employment of industry s in city i; $E_{i}$ is the total employment in city i; $E_{s\prime }$ represents the employment of industry s except city i; and $E^{\prime }$ is the total employment in the country other than city i.

For the measurement of the diversified agglomeration index, referring to the study of Han et al. (2015) [67], the modified Herfindahl–Hirschman Index concentration index was used, the formula for which is:

(8) $$div_{it}=\sum \frac{E_{is}}{E_{i}}\left\{\frac{1/\sum_{s\prime \neq s}^{n}{\left[E_{is\prime }/\left(E_{i}-E_{is}\right)\right]}^{2}}{1/\sum_{s\prime \neq s}^{n}{\left[E_{s\prime }/\left(E-E_{s}\right)\right]}^{2}}\right\}$$

where $E_{is\prime }$ denotes the employment of industry $s^{\prime }$ in city i other than industry s; $E_{s\prime }$ represents the employment in a national productive-service industry $s^{\prime }$ other than industry s; and the meanings of other variables are the same as above.

#### 3.2.3. Mediating/Moderating Variable

In this paper, industrial-structure upgrading (ind) is both a mediating variable and a moderating variable. Industrial-structure upgrading refers to the dynamic process of industrial-structure evolution from a low level to a higher level, which is mainly reflected in the advancement of industrial structure. By reference to Ma et al. (2018), the ratio of the added value of tertiary industry to the added value of secondary industry is used to measure the upgrading of industrial structure [68]. This indicator can reflect the degree of transformation of industrial structure from industrial-oriented to service-oriented structuration, and the larger the indicator, the higher the level of industrial-structure servitization and the more obvious the industrial-structure upgrade.

#### 3.2.4. Control Variables

In order to weaken the estimation bias caused by the omission of variables from the model and to identify the impact of productive-services agglomeration on the GTFPs of cities in the Yellow River Basin more accurately, the following control variables were added to the model in this paper: (1) science and technology input intensity (st), expressed as the proportion of a city’s science and technology expenditure relative to public expenditure; (2) the level of foreign direct investment (fdi), measured by the proportion of actual foreign direct investment utilized relative to GDP; (3) the level of financial development (fd), measured by the proportion of total loans from financial institutions relative to GDP at the end of the year; (4) information infrastructure (inf), measured by the number of households with Internet access; and (5) economic-development level (pgdp), measured using GDP per capita.

#### 3.2.5. Description of the Samples and Data

The samples selected in this paper were 68 prefectural-level-and-above cities in the Yellow River Basin from 2006–2019. The Yellow River Basin, in its physical geography, includes nine provinces, namely, Shandong, Henan, Shanxi, Shaanxi, Inner Mongolia, Ningxia, Sichuan, Gansu, and Qinghai provinces, and contains 79 cities (or autonomous prefectures and leagues) at prefecture level and above. Since the Yellow River flows through only two autonomous prefectures, Aba and Ganzi, in Sichuan Province, and the eastern part of Inner Mongolia is included in the Northeast Regional Revitalization Plan, the above regions are not included in this paper. In addition, limited by the availability of data for cities in Qinghai, Gansu, and Ningxia provinces, cities with serious data deficiencies were excluded, so that 68 cities at the prefecture level and above were finally identified as the research subjects. Table 1 reports the list of sample cities selected for our study.

All the raw data used in this paper are mainly from the China City Statistical Yearbook, the China Urban Construction Statistical Yearbook, statistical yearbooks and statistical bulletins of the relevant provinces and cities, and the EPS data platform. For missing data, the mean interpolation method was used to complete the process. Some variables involving price factors were deflated, with 2006 as the base period. Table 2 reports the descriptive statistical results for the variables in our model.

## 4. Results and Discussion

### 4.1. The Benchmark Estimated Results

To examine the direct impact of productive-services agglomeration on the GTFPs of cities in the Yellow River Basin, we used a dual fixed-effects model for estimations, and the regression results are shown in Table 3. Concretely, columns (1) to (4) in Table 3 report the impacts of specialized agglomeration and diversified agglomeration on city GTFPs in the Yellow River Basin. Regardless of whether the control variables were added or not, the regression coefficients of specialized agglomeration (lnspe) and diversified agglomeration (lndiv) were significantly positive at the level of 1%, indicating that the two different agglomeration patterns of productive services can significantly promote the growth of GTFP in the Yellow River Basin, which supports hypothesis H1. Columns (5) and (6) incorporate both specialized agglomeration and diversified agglomeration of productive services into the model. In the case of adding control variables, it can be seen that the influence coefficients of specialized agglomeration and diversified agglomeration on city GTFP in the Yellow River Basin are 0.064 and 0.049, respectively, with the former being significant at the 1% level and the latter significant at the 10% level, which means that, although both specialized agglomeration and diversified agglomeration can significantly contribute to city GTFP in the Yellow River Basin, the promoting effect of specialized agglomeration is greater than that of diversified agglomeration.

In the Yellow River Basin, why is specialized agglomeration beneficial in promoting GTFP? We believe the following explanation is possible. The level of economic development in the Yellow River Basin is relatively low, with primary processing industries and energy-heavy industries being the main types of industry, which has led to a lack of innovation vitality and serious pollution emissions in the development process. On the one hand, the economies of scale and knowledge spillover caused by specialized agglomeration can effectively drive the integration of various factors, reduce the space–time cost of sharing information and production materials among enterprises of the same type, and enhance the innovation vitality of enterprises. On the other hand, the scale effect of specialized agglomeration in the process of pollution treatment can not only realize the centralized treatment of pollutants but also effectively reduce the cost of pollution treatment for enterprises, thus curbing environmental pollution. Given the above two points, specialized agglomeration becomes an effective means to promote city GTFP in the Yellow River Basin.

Diversified agglomeration also helps to improve city GTFP in the Yellow River Basin, but the effect is weaker than that of specialized agglomeration. The possible reasons are as follows. Diversified agglomeration breaks through industry shackles and promotes exchange and cooperation among different types of enterprises, and the cross-border integration of knowledge and technology enhances inter-industry collaborative innovation capability, thus contributing to the improvement of the technological efficiency of enterprises in the agglomeration area. In addition, the cross-industry flow of labor forces with different knowledge reserves forms a diversified “knowledge reservoir”, which promotes the gradual movement of productive services to the middle and high end, thus helping to improve GTFP. However, currently the industrial structure of most cities in the Yellow River Basin is still low-end, and the demand for productive services is relatively monotonic, which greatly limits the positive externalities of the diversified agglomeration of productive services, resulting in the phenomenon that the promoting effect of diversified agglomeration on GTFP is weaker than that of specialized agglomeration.

### 4.2. Robustness Test

To ensure the reliability of the benchmark regression results, we adopted two approaches for robustness testing. First, the independent variables were lagged by one period, which also helped to alleviate the possible endogeneity of the model. The results are shown in column (1) of Table 4. Second, the data outliers were removed, and the model was re-estimated after performing a 5% winsorization of the sample. The results are shown in column (2) of Table 4. It can be seen that the coefficients of specialized and diversified agglomeration of productive services were still significantly positive under the two test methods, which is consistent with the benchmark results, indicating that the above results are robust and reliable.

### 4.3. Heterogeneity Analysis

#### 4.3.1. Regional Heterogeneity

Due to the large differences in endowment conditions and economic-development levels between upstream cities and middle and downstream cities in the Yellow River Basin, there may be regional heterogeneity in the impact of productive-services agglomeration on GTFP. Therefore, we conducted group regressions on the upstream cities and the middle and downstream cities to examine the differences in the impacts of productive-services agglomeration on city GTFPs in different regions. The results are shown in columns (1) to (4) of Table 5. We focus on the interpretation of the results with the inclusion of control variables. Column (2) shows that both specialized agglomeration and diversified agglomeration can significantly improve the GTFPs of the upstream cities in the Yellow River Basin. The disadvantageous geographical location of upstream cities, coupled with imperfect infrastructure, makes industrial development inadequate. The agglomeration of productive services in the upstream area can serve to improve the existing inadequate development conditions, thus promoting GTFP. For cities in the middle and downstream of the Yellow River Basin, it can be seen from column (4) that diversified agglomeration can significantly promote GTFP, but the promoting effect of specialized agglomeration is not significant. This may be due to the fact that cities in the middle and downstream have relatively better geographical locations, transportation bases, and factor-endowment conditions, and that the specialized agglomeration of productive services started earlier and has already entered a mature stage. With rapid economic development, the market sizes of middle and downstream cities are increasing, and the demand for productive services tends to be diversified.

#### 4.3.2. City-Size Heterogeneity

Considering that the impact of productive-services agglomeration on GTFP is also affected by city size [69], we further divided the sample cities into large cities and small and medium cities to examine the heterogeneity of the impact of productive-services agglomeration on GTFP in cities of different sizes, and the results are presented in columns (5) to (8) of Table 5. From columns (5) and (6), it can be seen that both specialized and diversified agglomeration of productive services have non-significant negative effects on GTFP in large cities. A possible reason is that most of the cities with larger population sizes in the Yellow River Basin are provincial capitals with relatively well-developed infrastructures, high-quality public services, and larger market potentials, such that industrial agglomeration in large cities reaches a higher level. However, due to the low level of comprehensive bearing capacity of cities in the Yellow River Basin, the large number of enterprises in the productive services leads to the transformation of the economies of scale generated by the early agglomeration according to the “crowding effect” and to the phenomenon of diseconomies of scale, which inhibits the improvement of GTFP. Moreover, the concentration of a large number of enterprises in large cities is likely to put greater pressure on local resources and environment, leading to the emergence of negative externalities of agglomeration, thus hindering green development. In columns (7) and (8), we provide the results on the impact of productive-services agglomeration on GTFP in small and medium-sized cities in the Yellow River Basin. The regression coefficients show that only specialized agglomeration can improve GTFP, while the promoting effect of diversified agglomeration is not significant. The reason may be that the market sizes of small and medium-sized cities in the Yellow River Basin are small and the demand for specialized agglomeration of productive services is more urgent, while diversified agglomeration has not yet developed at scale.

### 4.4. Analysis of Influencing Mechanisms

#### 4.4.1. Mediating-Effect Test

Table 6 reports the mediating-effect test results for the influence of productive-services agglomeration on city GTFP in the Yellow River Basin through industrial-structure upgrading. The regression results in columns (1) and (2) show that industrial-structure upgrading has a significant intermediating effect on the specialized agglomeration of productive services affecting city GTFP in the Yellow River Basin. This indicates that specialized agglomeration has a positive driving effect on the upgrading of industrial structure in the Yellow River Basin, which, in turn, promotes GTFP. The probable reason is that the specialized agglomeration of productive services brings together a large amount of high-quality human capital and technological factors, which facilitate exchange and cooperation among enterprises and promote industrial technological progress and industrial-structure upgrading, thus better optimizing resource allocation and enhancing GTFP in the Yellow River Basin. However, the imbalances between and unreasonable industrial structures among cities in the Yellow River Basin may weaken the promotion of industrial-structure upgrading on GTFP in the short term. From columns (3) and (4), it can be seen that the diversified agglomeration of productive services does not significantly contribute to the industrial-structure upgrading of cities in the Yellow River Basin. Therefore, there is no significant mediating effect of industrial-structure upgrading in the process of diversified agglomeration to enhance city GTFP in the Yellow River Basin, and the Sobel test is consistent with the above findings. This may be due to the low-level redundant construction of the diversified agglomeration of productive services in cities in the Yellow River Basin, which reduces the efficiency of resource utilization and is not conducive to promoting the upgrading of industrial structure. Based on the above analysis, the mediating-effect test results validate hypotheses H2, H3, and H4 proposed in Section 2.

#### 4.4.2. Moderating-Effect Test

Table 7 reports the test results for the moderating effect of industrial-structure upgrading on the process of productive-services agglomeration affecting city GTFP in the Yellow River Basin. The regression results in column (1) show that the effect of specialized agglomeration on city GTFP in the Yellow River Basin is significantly positive, and the coefficient of the interaction term (ln*spe* × ln*ind*) is −0.145 and passes the significance test at the 1% level, which indicates that industrial-structure upgrading has a significant negative moderating effect on the specialized agglomeration of productive services and city GTFP in the Yellow River Basin, such that hypothesis H5 is confirmed. According to column (2), it can be seen that the effect of diversified agglomeration on GTFP is significantly positive, but the coefficient of the interaction term (ln*div* × ln*ind*) is not significant, which indicates that industrial-structure upgrading does not have a moderating effect on the process of diversified agglomeration affecting city GTFP in the Yellow River Basin.

There are three possible reasons for the above results. Firstly, most cities in the Yellow River Basin are dominated by resource-based industries and traditional manufacturing industries, and industrial development is highly dependent on resources. In the process of transformation and upgrading, industries face the double dilemma of external conditions and insufficient endogenous motivation, and traditional technological progress has not truly realized greenization, such that the upgrading of industrial structure at this stage is not conductive to the improvement of GTFP. Secondly, under the influence of the industrial policy of “retreating from the secondary industry to the tertiary industry”, although the proportion of tertiary industries in the Yellow River Basin relative to secondary industries has increased, the level of industrial-structure optimization in most cities is not high, and they are still in the transition period of industrial transformation. Most of the transferred industries are low-end, resulting in low levels of economic development. Finally, in the context of the development of the specialized agglomeration of productive services, the rapid expansion of the production capacities of cities has sharply increased the demand for energy. However, due to the inadequate economic development, environmental regulation, and low industrial ecological levels of cities in the Yellow River Basin, emission intensities for the industrial “three wastes” have not been effectively reduced. The lack of necessary industrial links between cities and rural areas has led to “island effects”, which further aggravate the pollution in some areas.

## 5. Conclusions

In this paper, we used the dual fixed-effects model to examine the impact of productive-services agglomeration on GTFP based on panel data for 68 prefectural-level-and-above cities in the Yellow River Basin from 2006 to 2019. Moreover, the mediating-effect model and the moderating-effect model were used to test the role of industrial-structure upgrading in the process of productive-services agglomeration affecting GTFP. The main conclusions are as follows. First, both specialized agglomeration and diversified agglomeration of productive services can significantly improve the GTFPs of cities in the Yellow River Basin, and the growth effect of specialized agglomeration on GTFP is greater than that of diversified agglomeration. Second, regional heterogeneity tests showed that diversified agglomeration can significantly promote GTFP growth in mid–downstream cities, while the effect of specialized agglomeration is not obvious. For upstream cities, both specialized agglomeration and diversified agglomeration exert significant GTFP growth effects. Third, the analysis of city-size heterogeneity showed that specialized agglomeration has a significant positive effect on the promotion of GTFP in small and medium-sized cities, while diversified agglomeration does not exert such an effect. For large cities, neither of the two productive-services-agglomeration patterns has a significant effect on GTFP in the Yellow River Basin cities. Fourth, the mediating-effect and moderating-effect tests showed that specialized agglomeration can promote the GTFPs of cities in the Yellow River basin by promoting industrial-structure upgrading, while diversified agglomeration does not have this transmission mechanism. Meanwhile, industrial-structure upgrading plays a negative moderating role in the process of specialized agglomeration affecting the GTFPs of cities in the Yellow River Basin but does not have a moderating effect on the impact of diversified agglomeration.

Based on the above findings, to further promote green development and improve GTFP in the Yellow River Basin, we argue that it is necessary to accelerate the upgrading of industrial structure and strengthen the high-end agglomeration of productive services. At the same time, the central government should guide productive services to form reasonable agglomeration patterns in different zones and cities of different sizes according to the resource endowments of different cities. The specific recommendations are as follows:

(1) The upstream cities should fully consider their ecological protection responsibilities, reasonably plan the agglomeration areas rely on local resource endowments and advantageous industries, introduce and undertake high quality enterprises, such as R&D design and modern logistics in orderly manner, improve the technical added value and ecological value of products.
(2) The middle and downstream cities should make full use of the advantages of the diversified agglomeration of productive service industries, actively promote diversified development and the high-quality agglomeration of productive-service industries, and avoid the problem of low-level duplication of construction. In addition, the middle and downstream cities should guide the coordinated development of high-end and low-end productive services, so as to form benign cooperation and competition mechanisms among enterprises in the agglomeration area and promote the transformation of low-end productive services to high-end services.
(3) Large cities should accelerate the development of knowledge and technology-intensive high-end productive services that rely on talent and technology advantages. Small and medium-sized cities should fully consider limitations of city size and avoid blindly pursuing high-end and diversified development of industries. Instead, they should focus on promoting the specialized agglomeration of low-end productive services and improving the efficiency of resource allocation.
(4) The central government should encourage large cities in the Yellow River Basin to help small and medium-sized cities. By promoting the transfer of low-end productive services to alleviate pressures on resources and the environment caused by the excessive agglomeration of productive services in large cities, the positive spatial externality of productive services agglomeration can be released.

Though we have carried out work on the research theme, there are still some limitations that it will be necessary to improve on in future research.

Firstly, there are limitations with respect to the sample data. Since the latest statistics at the city level in China have not been fully published, it is difficult for us to ensure the timeliness of the data. In future studies, the time span of the samples can be extended with updated data and a more rigorous dynamic econometric model can be constructed for empirical studies. In addition, due to data being missing for some indicators in the cities upstream of the Yellow River Basin, we mainly used the linear interpolation method to solve this problem. Although this can meet research needs, errors in data measurement may still interfere with the results. Therefore, in future research, scholars need to focus on the quality of sample data.

Secondly, the industry divisions are not detailed enough. In this paper, we mainly used different agglomeration patterns as the theoretical bases to study the effects of the specialized agglomeration and diversified agglomeration of productive services on GTFP in the Yellow River Basin. However, due to the vague classification criteria and the limitations of the sample data, we did not make a detailed classification of productive services, which also leaves room for future improvement. Scholars could further classify productive services into high-end and low-end productive services and explore the differential effects of the two on GTFP in the Yellow River Basin.

Thirdly, the mechanistic analysis needs to be expanded. In this paper, we analyzed the influence of productive-services agglomeration on GTFP in the Yellow River Basin from the perspective of industrial-structure upgrading, but it is obvious that this is not the only transmission mechanism. The agglomeration of productive services may also enhance regional green TFP through curbing pollution emissions, improving resource-allocation efficiency, and promoting green innovation. Therefore, scholars can focus on the theoretical and empirical analysis of the above three mechanisms to enrich the existing research results.

## Figures and Tables

**Table 1:** List of the sample cities.

	Cities
Upstream (21)	Xining, Yinchuan, Shizuishan, Wuzhong, Guyuan, Lanzhou, Baiyin, Tianshui, Wuwei, Zhangye, Pingliang, Jiuquan, Qingyang, Dingxi, Hohhot, Baotou, Wuhai, Chifeng, Erdos, Bayannaoer, Wulanchabu
Midstream (20)	Taiyuan, Datong, Yangquan, Changzhi, Jincheng, Shuozhou, Jinzhong, Yuncheng, Xinzhou, Linfen, Lvliang, Xi’an, Tongchuan, Baoji, Xianyang, Weinan, Yan’an, Hanzhong, Yulin, Shangluo
Downstream (27)	Zhengzhou, Kaifeng, Luoyang, Anyang, Hebi, Xinxiang, Jiaozuo, Puyang, Sanmenxia, Shangqiu, Zhoukou, Zhumadian, Jinan, Qingdao, Zibo, Zaozhuang, Dongying, Yantai, Weifang, Jining, Taian, Weihai, Rizhao, Dezhou, Liaocheng, Binzhou, Heze

**Table 2:** Summary statistics for the variables.

Variable	Obs	Mean	Sd	Median	Max	Min
*gtfp*	952	0.892	0.279	0.844	3.779	0.590
*spe*	952	0.058	0.023	0.055	0.176	0.016
*div*	952	0.222	0.097	0.207	0.956	0.062
*ind*	952	0.880	0.458	0.776	4.107	0.194
*st*	952	0.012	0.018	0.009	0.362	0.001
*fdi*	952	0.013	0.016	0.008	0.207	0.000
*fd*	952	0.908	0.741	0.671	9.622	0.222
*inf*	952	61.190	73.670	37.610	697.000	1.500
*pgdp*	952	43,428	33,081	35,105	256,877	2767

**Table 3:** The benchmark estimated results.

Variable	ln*gtfp*					
	(1)	(2)	(3)	(4)	(5)	(6)
ln*spe*	0.083 ***	0.081 ***			0.068 ***	0.064 ***
	(0.020)	(0.020)			(0.022)	(0.023)
ln*div*			0.080 ***	0.086 ***	0.041	0.049 *
			(0.025)	(0.026)	(0.028)	(0.029)
*constant*	0.103 *	−0.442	−0.011	−0.529	0.126 **	−0.382
	(0.059)	(0.410)	(0.041)	(0.409)	(0.061)	(0.411)
*Controls*	No	Yes	No	Yes	No	Yes
City FE	Yes	Yes	Yes	Yes	Yes	Yes
Year FE	Yes	Yes	Yes	Yes	Yes	Yes
N	952	952	952	952	952	952
R^2^	0.476	0.481	0.472	0.477	0.477	0.482

Notes: Standard errors in parentheses; ***, **, and * represent significance levels at 1%, 5%, and 10%.

**Table 4:** The robustness test results.

Variable	ln*gtfp*	
	(1)	(2)
L.ln*spe*	0.050 **	
	(0.023)	
L.ln*div*	0.051 *	
	(0.029)	
ln*spe*		0.064 ***
		(0.022)
ln*div*		0.049 *
		(0.029)
*constant*	−0.572	−0.374
	(0.435)	(0.406)
*Controls*	Yes	Yes
City FE	Yes	Yes
Year FE	Yes	Yes
N	884	952
R^2^	0.485	0.482

Notes: Standard errors in parentheses; ***, **, and * represent significance levels at 1%, 5%, and 10%.

**Table 5:** The heterogeneous regression results.

Variable	Upstream Cities		Mid–Downstream Cities		Large Cities		Mid–Small Cities	
	(1)	(2)	(3)	(4)	(5)	(6)	(7)	(8)
ln*spe*	0.125 ***	0.132 ***	0.019	0.011	−0.004	−0.005	0.113 ***	0.111 ***
	(0.048)	(0.050)	(0.024)	(0.024)	(0.028)	(0.029)	(0.032)	(0.033)
ln*div*	0.145 *	0.146 *	0.040	0.046 *	−0.052	−0.061	0.045	0.051
	(0.077)	(0.084)	(0.027)	(0.028)	(0.044)	(0.047)	(0.037)	(0.039)
*constant*	0.538 ***	−0.249	−0.047	−0.881 *	−0.262 **	−0.683	0.271 ***	−0.446
	(0.150)	(0.922)	(0.061)	(0.451)	(0.106)	(0.840)	(0.078)	(0.491)
*Controls*	No	Yes	No	Yes	No	Yes	No	Yes
City FE	Yes	Yes	Yes	Yes	Yes	Yes	Yes	Yes
Year FE	Yes	Yes	Yes	Yes	Yes	Yes	Yes	Yes
N	294	294	658	658	294	294	658	658
R^2^	0.509	0.512	0.435	0.446	0.338	0.353	0.514	0.520

Notes: Standard errors in parentheses; ***, **, and * represent significance levels at 1%, 5%, and 10%.

**Table 6:** Results of the mediating-effect test.

Variable	Specialized Agglomeration		Diversified Agglomeration	
	(1)	(2)	(3)	(4)
	ln*ind*	ln*gtfp*	ln*ind*	ln*gtfp*
ln*spe*	0.042 **	0.077 ***		
	(0.018)	(0.020)		
ln*div*			0.021	0.084 ***
			(0.023)	(0.025)
ln*ind*		0.107 ***		0.115 ***
		(0.037)		(0.037)
*constant*	6.174 ***	−1.092 **	6.071 ***	−1.216 ***
	(0.368)	(0.465)	(0.367)	(0.462)
*Controls*	Yes	Yes	Yes	Yes
City FE	Yes	Yes	Yes	Yes
Year FE	Yes	Yes	Yes	Yes
N	952	952	952	952
R^2^	0.930	0.485	0.929	0.483
Sobel test		0.004 *		0.002
		(0.002)		(0.002)
Proportion of mediating effect		6%		/

Notes: Standard errors in parentheses; ***, **, and * represent significance levels at 1%, 5%, and 10%.

**Table 7:** Results of the moderating-effect test.

Variable	Specialized Agglomeration	Diversified Agglomeration
	(1)	(2)
	ln*gtfp*	ln*gtfp*
ln*spe*	0.062 ***	
	(0.020)	
ln*div*		0.061 **
		(0.030)
ln*ind*	−0.301 ***	0.025
	(0.108)	(0.070)
ln*spe* × ln*ind*	−0.145 ***	
	(0.036)	
Ln*div* × ln*ind*		−0.062
		(0.041)
*constant*	−1.216 ***	−1.303 ***
	(0.462)	(0.465)
*Controls*	Yes	Yes
City FE	Yes	Yes
Year FE	Yes	Yes
N	952	952
R^2^	0.495	0.485

Notes: Standard errors in parentheses; *** and ** represent significance levels at 1% and 5%.

## Data Availability

Readers can obtain the raw datasets used in this paper by themselves through the data sources described in Section 3, or by contacting the first author or the corresponding author.
